# The pragmatic, rapid, and iterative dissemination & implementation (PRIDI) cycle: Adapting to the dynamic nature of public health emergencies

**DOI:** 10.21203/rs.3.rs-188929/v1

**Published:** 2021-02-09

**Authors:** Reza Yousefi Nooraie, Rachel C Shelton, Kevin Fiscella, Bethany M Kwan, James M McMahon

**Affiliations:** University of Rochester School of Medicine and Dentistry; Columbia University Mailman School of Public Health; University of Rochester School of Medicine and Dentistry; University of Colorado Anschutz Medical Campus; University of Rochester School of Nursing

**Keywords:** rapid-cycle, public health emergencies, D&I models, COVID-19

## Abstract

**Background:**

Public health emergencies – such as the 2020 COVID19 pandemic –accelerate the need for both evidence generation and rapid dissemination and implementation (D&I) of evidence where it is most needed. In this paper, we reflect on how D&I frameworks and methods can be pragmatic (i.e., relevant to real-world context) tools for rapid and iterative planning, implementation, evaluation, and dissemination of evidence to address public health emergencies. **The Pragmatic, Rapid, and Iterative D&I (PRIDI) Cycle**: The PRIDI Cycle is based on a “double-loop” learning process, reflecting the iterative and adaptive D&I, along with iterative re-consideration of goals and priorities, interventions and corresponding D&I strategies, and needs and capacities of individuals and contexts. Stakeholder engagement is essential- which itself is an evolving activity. The results of iterative evaluations should be communicated with local implementers and stakeholders through customized feedbacks.

**Conclusion:**

Even when the health system priority is provision of the best care to the individuals in need, and scientists are focused on development of effective diagnostic and therapeutic technologies, planning for D&I is critical. Without a flexible and adapting process of D&I, which is responsive to emerging evidence generation cycles, and is closely connected to stakeholders and target users through engagement and feedback, the interventions to mitigate public health emergencies – such as the COVID19 pandemic - will have limited reach and impact on populations that would most benefit. The PRIDI cycle is intended to provide a pragmatic approach to support planning for D&I throughout the evidence generation process.

## Background

Public health emergencies – such as the current COVID19 pandemic – dramatically accelerate the need for evidence generation and synthesis, as well as the rapid dissemination and implementation of evidence-based practices and interventions. (1–3) In a matter of weeks in late winter 2020, the scientific enterprise in clinical and translational research in public health and medicine was nearly universally re-oriented to pressing and emergent COVID19-related concerns. In addition to research on tests and treatments, there is a need for studying emerging healthcare system-level interventions. From the rapid adoption of telehealth care across nearly every health discipline, (4–6) development and implementation of procedures for risk stratification and delaying elective procedures during pandemic, (7) to strategies to re-open and revamp health care and messaging considering physical distancing principles, (8, 9) the pandemic is driving rapid change in health care and public health systems.

Dissemination and implementation (D&I) science has emerged as an evolving field to address the well-documented gap between research and practice. (10) Dissemination specifically relates to the active or planned communication of best practices/interventions to encourage their widespread adoption among key decision-makers across a range of settings, whereas implementation focuses on factors and strategies to support the adaptation and the routine use and delivery of the recommended practices or evidence-based interventions in real-world clinical and community settings. (11) The D&I of organizational- and system-level interventions, practices, or policies often involve modifying existing structures (physical or technological), redesigning processes of work and clinical workflow, and redefining roles, operating within a broader complex and dynamic organizational or healthcare system context. In emergencies, these already challenging changes can become even more burdened by strained resources, competing demands, and overextended or strained systems and healthcare workers. Decision-makers may think of systematic planning for D&I as expendable or irrelevant in emergencies – perhaps perceived as an academic exercise or too time consuming with limited added value. However, unsuccessful or inequitable implementation of resource-intensive system-level interventions can result in treatment delays, inequities in access to and delivery of care and poor population health outcomes – including death. For example, the unequitable delivery of care may partially explain the racial and ethnic disparities in COVID19 mortality; (12, 13) or delays in provision of diagnostic tests may reduce the effectiveness of contact-tracing strategies. (14)

However, classical D&I frameworks and approaches may need some rapid adaptations to be pragmatic and of most use in rapidly evolving emergency situations. An important feature of emergencies is the quick, dynamic, and unpredictable course of events, (15) which makes planning for D&I challenging. Some examples are:

- The **health problem** itself may be dynamic and rapidly changing. In February 2020, the main concern of many health systems was implementing case finding and quarantine strategies, while in March 2020, it was allocating ICU beds and ventilators, and in May 2020, safe strategies to gradually lifting lock downs. (16)
- The **evidence** and associated interventions or solutions and strategies to support delivery of evidence are not fixed, as the evidence for effectiveness of cloth masks, hydroxychloroquine, antibody tests, and various diagnostic approaches has evolved rapidly. (17) The COVID19 infodemic resulted in the outpouring of misinformation, which complicated the separation of fact from fiction(18) and contributed to confusion in messaging among the public, as well as trust of the information provided.
- The **contexts/settings** in which COVID19 is being transmitted and in which testing and treatment are occurring are not fixed. Both inner settings (hospital resources, hospital policies, capacity, exhaustion)(19, 20) and outer settings (effective social distancing, economical constraints, state/national policies) (21, 22) are changing by day and over time, and require constant monitoring and reconsideration of plans.
- **Key stakeholders (e.g. healthcare workers, patients, community members, leadership)** within systems and broader communities have evolving concerns, needs, and values. Their readiness, knowledge, and capabilities are evolving based on changing circumstances and contexts, and their trust of medical institutions and perceptions of the importance of scientific evidence varies.
- There is usually **redundancy** and **parallelism** within systems, which positively and negatively affects the implementation of evidence-based processes and practices. On the positive side, we can learn from the experience of other hospitals who deal with similar situations and challenges (e.g. in allocating ventilators and ICU beds). (23) On the negative side, redundancy and parallelism and lack of communication may result in confusion, conflicts, dilution of resources, and burn-out, and lack of monitoring and evaluation of what practices are both feasible and have impact.
- Additional complexities that need to be addressed include the striking racial/ethnic inequities that have been apparent with respect to COVID19 morbidity and mortality, related in part to embedded systems that create and reinforce structural and interpersonal forms of discrimination and racism. (12, 24)

To align both the science of D&I with the practice of D&I in real-world settings, it is important to explicate how health systems can apply D&I frameworks and methods rapidly, effectively, equitably, and with few resources to guide local adoption of evidence-based interventions or emerging best practices/protocols (informed by the best available evidence at the time). In this paper, we reflect on widely adopted D&I frameworks and tools and how they should be adapted to address dynamic trajectories of emergency situations.

## The Pragmatic, Rapid, And Iterative D&i(pridi) Cycle

Figure 1 shows the PRIDI model for D&I. It depicts the dynamic connection between the cyclical process of executing and evaluating D&I (center), the intervention and strategies (left side), the multi-level nature of the context (upper side), and goals and outcomes of D&I (right side). Consistent with recent emphasis on iterative and pragmatic nature of D&I, (25, 26) its journey is not a linear process, particularly in the fluid and dynamic contexts of emergencies. This cyclical process of Assess > Plan > Do > Evaluate > Report should be done quickly and iteratively as an intervention and strategies to support it are rolled out. (27)

As shown in Fig. 1, we incorporated this cyclical process to the center of the PRIDI model. The cycle of D&I itself activates lateral cycles (shown by the cyclical relation between the middle circle and three arms), which involve revisiting the mental models, goals and outcomes, interventions and D&I strategies, and individuals and contexts, through the course of D&I cycles. It resembles a double-loop learning model. (28, 29) This is particularly critical in emergencies, where traditional mental models may not fit the emerging problems and contexts. If we apply Plan > Do > Study > Act (PDSA) cycles using existing models i.e. single loop learning, we might fail to learn from the higher order feedback loops that requires more than incremental improvements in efficiency and time. Second order learning might inform entirely different approaches based on different assumptions and different mental models.

To the extent possible, monitoring and evaluation should be prioritized, and results should be regularly communicated with stakeholders, and meaningfully and consistently incorporated in any re-design or planned adaptations/modifications within the system. (2) If an intervention or a D&I strategy is not working, it should be modified or abandoned (de-implemented) in a timely manner. Evaluations and monitoring may include information that changes the nature of the evidence supporting the effectiveness of the intervention itself or strategies to support its use (see cyclical path from the implementation to effectiveness).

The engagement of stakeholders within these dynamic contexts is critical throughout this process to understand what is working or not and why, where inequities are emerging, and the feasibility and acceptability of the programs and practices. The double loop nature of the process also has implications for engagement of diverse stakeholders in the context of psychological safety where people feel free to express contrarian views thus fostering opportunities to challenge conventional assumptions. For example, suppose we assume that African Americans by virtue of higher SARS-CoV-2 infection rates and worse outcomes will have higher demand for COVID-19 testing and vaccines in development. This would be a reasonable assumption from which we could develop cyclical PDSAs for messaging. This assumption would suggest that finding ways to promote awareness in the African American community regarding where to get tested and where to receive the future vaccine will reduce disparities in infection. Yet, if the African American community were at the table and divergent views were encouraged based on recognition of second order learning, members might express reservations about COVID-19 testing including risk for family separation, forced quarantine with pay, and greater stigma. Similarly, members might voice deep skepticism towards receiving future vaccines including mistrust of government statements, concerns about being guinea pigs for a vaccine that has been rushed to market, and/or concerns about the vaccine containing virus. This second order learning might suggest a fundamentally different approach rather than incremental changes in content, dose or frequency in messages.

In Table 1, we summarized the suggested aspects of D&I activity that should be collected, discussed, and re-evaluated at each round.

## Interventions And Strategies

Consistent with RE-AIM/PRISM model (30), the interventions and strategies to facilitate their dissemination, adoption, and use are the key elements of D&I efforts, that are shown in the left side of Fig. 1:

- The **intervention (e.g. evidence-based practice, policy, program, treatment)** to be disseminated and implemented (e.g. establishing a call center for follow-up of patients in home-isolation, setting up an online meeting model for grand rounds, an online screening tool for self-assessment of symptoms, best practices for mental health screening among COVID19 patients/healthcare workers, safety protocols and policies for birthing mothers) (e.g. the ‘what’)
- **D&I strategies** involve the processes, approaches, or interventions that facilitate and enhance the proactive dissemination and implementation of the interventions. Examples include tailored email/online communication for the self-assessment platform, literacy appropriate instructional packages for patients about the COVID19 call center, staff training/education to learn about the new workflow, motivational incentives to enhance staff participation in grand rounds. See Powell et al. (2015) for full ERIC taxonomy of implementation strategies. (31)

Interventions generally include a core (the essence or function of the intervention that is responsible for its impact), and an adaptable periphery (that could be modified to adapt to various contexts and situations). (32) Ignoring the distinction of these two components may result in rigid interventions that are not flexible to survive varying and unprecedented contextual variations and barriers, or that are too complex or costly to be implemented. As such, it is important that a flexible approach is taken during the design of D&I activities, and the local implementers are trusted to adapt the intervention to fit into their own local contexts, resources, needs, and policies. Consequently, we added Adaptation as an important phase in PRIDI Cycle (Center of Fig. 1).

D&I adaptation models may be useful to help guide planned adaptations (e.g. ADAPT-ITT)(33), to help balance considerations of fit and fidelity. Ideally, the core component of the intervention should be defined, dynamically updated (as changes are made over time), and communicated; relevant data could be collected through iterative evaluations to understand the impact of both the core elements of the program, any planned adaptations made, as well as evolutions of the program made across its life course. (34) For example, preventive health messages delivered through health organizations such as the Centers for Disease Control and Prevention (CDC)(35) and local and state health authorities typically target broad audiences and are not always adapted to the needs, values, or expectations of vulnerable individuals and communities. The messages may not address the limited behavioral control of the target audience (e.g. in practicing social distancing or staying at home), may not include information about local services and resources, and may not be adapted to the literacy levels of individuals who may be at greatest risk for COVID-19. (36, 37) For example, an individual living in a dense, multi-generational household may have difficulty following isolation and physical distancing guidelines or lack digital technology to access functional health literacy resources. (38, 39) Communities of color, including Black Americans who have experienced striking COVID-19 inequities, are more likely to be exposed to multiple layers of structural racism, including living in buildings and neighborhoods that are more crowded and have poorer infrastructures, irrespective of income, and may feel unsafe using face masks in public. Asian or Asian Americans may face stigma related to the disease due to misinformation about its origins and spread. An individual living in unstable economic conditions who needs access to employment may not be able to self-isolate for the recommended period of time while symptomatic. Therefore, standard messaging should be adapted to the needs, expectations, and capacities of diverse sub-groups and populations to be able to educate or motivate and improve the understanding of COVID-19 and both individual and community responses to it.

## Goals & Outcomes

Evaluation is not a one-time post-intervention process in D&I; it is an iterative, ongoing process that can enhance and inform the evolvability of evidence-based interventions and strategies, including their design, adaptation, refinement, and delivery throughout the process of D&I. Consequently, intended goals and outcomes of D&I should ideally be incorporated from the beginning (right side of Fig. 1). In emergency planning, the value of learning from continuous evaluation is even more essential, as the path forward can be more uncertain, the interventions are more experimental and their evidence-base evolving, and the clinical situation and healthcare contexts can change quickly. As such, it may be useful for decision-makers to have a compass to guide them as to whether they are moving in the right direction or need to re-assess and re-design and challenge existing models that might not fit with such a dynamic context.

RE-AIM provides a systematic conceptual framework to guide the planning, adaptation, and evaluation of the D&I activities, programs, practices or policies.(25, 40) An intervention should: Reach the target populations equitably (Did we reach the those who needed the intervention or would benefit the most from it?); be Effective (Did the intervention achieve its goals and impact on health behaviors/outcomes?); be widely Adopted (Did the settings and stakeholders/decision-makers adopt the intervention?); be Implemented (Did the target users or implementers actually use it as it was intended? How was it adapted?); and be Maintained/Sustained (Did the target users continue using it over time and did it continue to have long-term impact?). Importantly, in light of dynamic contexts, (41) RE-AIM can be iteratively applied to track these D&I indicators to help document where inequities and challenges in each of these areas are arising and to inform refinements of adaptations to respond to changing system challenges (e.g. costs, resources), population needs/values, and evolving evidence. (25)

Glasgow et al. (2020) applied RE-AIM iteratively in a participatory process to support prospective adjustments during implementation projects. (26) Through this cyclic process, it may be useful for implementing agents/teams to receive practical and customized feedback about their performance, so they can understand progress in comparison to the original goals or in comparison to other implementers in their setting, and correct their path if needed. (42) RE-AIM dimensions may differ in terms of importance and feasibility to assess. At each round of the cycle, stakeholders can decide which RE-AIM dimensions are more important, more in need of improvement, and more feasible to assess. (26)

## Individuals And Contexts

The upper side of Figure 1 shows the multi-layer and complex nature of contextual factors and their role in determining the success or failure of D&I efforts. It is critical to consciously consider the complexity of personal, inter-personal, organizational, social, economic, policy, community, and cultural contexts at the design phase, and across the continuous process of re-evaluation and adaptations throughout implementation phases. A seemingly useful intervention may fail to implement, since patients may find it irrelevant to their needs and characteristics, or may face certain financial and structural/logistical barriers to access and use it, or may not trust the source of the intervention; staff or administrators may find it burdensome (since many staff who are running these programs are delivering them in addition to their normal workload, they may be overwhelmed or have many competing demands under limited resources); at the organizational level, infrastructure needed to deliver the program may have geographical, demographic, and structural limitations. External environment factors, such as policies, economic challenges, and cultures and social norms are also rapidly changing. For example, adherence to long-term physical/social distancing may vary based on demographics and cultural backgrounds; (43) Country-level and state-level disease mitigation policies may affect the implementation and sustainment of interventions; (44) and wider economic impact of the lockdowns and current mitigation strategies may affect the effectiveness and sustainment implementation of those mitigation strategies (45) (through activation of feedback loops). Many of these barriers are difficult to overcome in emergency situations; however, having the tools to address them may facilitate development of innovative alternative solutions and enhance the reach and impact of evidence-based solutions, particularly with an eye towards health equity.

### Stakeholder engagement:

It may seem like an inappropriate time to engage stakeholders in the context of emergency situations. However, even brief engagement of stakeholders has immense benefits that make it worth considering, at the design phase and through the cyclical process of re-evaluation and re-design. (46) Stakeholders that are actively involved and engaged in the processes of dissemination and implementation may: (47)

- Feel more invested to help disseminate, implement, and sustain it
- Are prepared cognitively and operationally and are more committed to execute it
- May identify setting or culturally specific barriers that may have been have missed
- Provide real time feedback on whether strategies are working and inform important refinements or adaptations of interventions and strategies
- Enhance relevance and fit, and may propose innovative solutions that haven’t been considered

Stakeholder engagement may be applied at different degrees along the spectrum of implementation, depending on the availability of time and resources, and the nature of the intervention and D&I strategies. (48) Even at its lowest degrees (i.e. information provision and consultation) it can facilitate preparedness and elicitation of some feedback that may be critical in the success of D&I efforts. Given its key importance in informing the process of D&I, we showed ‘stakeholder engagement’ as a circle surrounding all phases of D&I cycle in PRIDI model (Fig. 1).

## Leadership

All mentioned processes are only possible under the context of strong organizational commitment, (49) as well as transformational (inspiring and motivating) and transactional (providing contingent rewards) leadership, (50) that have shown to predict implementation success. (51) Organizational leaders can help maximize the fit between all aspects of D&I activities (30), make and effectively communicate strategic decisions, and are nimble and ready to change course midway if the iterative evaluations suggest the need for modification of goals and strategies. A successful crisis leader (52) should be well-versed with the subject matter (e.g. public health) or consult team members with expertise in the specific area; should make evidence-based and timely decisions, while constantly collecting data from the environment; should inspire trust and confidence; and should feel responsible for the safety and welfare of the team members. In emergency situations it is very likely that multiple groups try independently to develop solutions, which may result in fragmented efforts and confusion. The leader should develop an effective project management structure as well as an atmosphere in which teams and individuals have means and feel safe to express criticisms and suggest alternative solutions.

## Conclusions

In this paper, we reflected on the cyclical model of ‘Assess > Plan > Do > Evaluate > Report’ (27), RE-AIM/PRISM framework (30), recent advancement of RE-AIM to incorporate equity (25), and to inform rapid implementation (26), and proposed PRIDI model that takes the dynamic nature of problems, interventions, evidence, contexts, and stakeholders into account. D&I in the context of emergency should be a continuous and iterative process. RE-AIM provides a framework for the evaluation of D&I activities, that includes **Reach**, **Effectiveness**, **Adoption**, **Implementation, Maintenance**. Recent extensions of this model can also inform more explicit consideration of understanding and addressing health equity and equitable implementation over time and in dynamic contexts. (25) Interventions are disseminated and implemented in complex and multi-layer contexts. Overlooking these complexities will hamper the success of the adoption, use, and impact of the intervention.

The cyclical process of D&I informs double loop learning processes, that may result in revisiting mental models, goals and outcomes, interventions and D&I strategies, and individuals and contexts. The results of cyclical evaluations should also be communicated with local implementers and stakeholders through customized, and actionable feedback. Stakeholder engagement is a key solution to understand and address contextual variations and barriers. It is a continuum ranging from informing the stakeholders to co-ownership, and will be critical to addressing some of the striking racial/ethnic and setting inequities evidence for COVID19, including redistribution of decision-making and resources with the community. The iterative process also accommodates for emergent evidence-generation and potential revisions in the evidence base of the interventions that are being implemented.

Even though the health system priority at this moment is provision of best care to the individuals in need and development of effective diagnostic and therapeutic technologies, (2) flexible and prospective planning for D&I is also critical. (2, 53) Without planning and tailoring, with the input and partnership of local stakeholders, D&I strategies will never reach target populations that would most benefit from it; will only be accessed and used by socio-demographic groups that face fewer structural barriers to care (hence deepening the equity gap); and will not sustain as intended. While limited organizational readiness and lack of time and resources are challenges to effective D&I plans, emergency response interventions may fail to meet their objectives and waste limited resources if critical D&I considerations are ignored. Development of infrastructures, organizational cultures, trainings, and establishment of processes towards a Rapid-Learning Health System (54, 55) and incorporation of D&I as its key component (55) will prepare healthcare systems and organizations to effectively respond to future emergencies. D&I methods and frameworks also need to adapt to the dynamic trajectories and complexity of emergency situations.

This paper calls for dynamic and adaptive D&I models that are responsive to rapid and unpredictable nature of emergencies through rapid and iterative cycles, continuous engagement of stakeholders, and incorporating the evolution of goals, interventions, and contexts. We also call for more dynamic and two-way translational dialogue between D&I and evidence generation research.

## Figures and Tables

**Table 1:** A template for recording progress in PRIDI cycles

Rounds		D&I goals	Intervention/Evidence	Intervention Adaptations/Refinements	Individuals: Users, D&I actors	Settings (Inner and Outer Context)	D&I strategies	Other Key Stakeholders	D&I/Effe Evaluatio Metrics (domain focus on
Round 1	Description								
	Opportunities and challenges								
	Plans for next round/plans to address challenges								
Round 2…	Progress/								
	Adaptations/Revisions								
	Opportunities and challenges								
	Plans for next round/plans to address challenges								

## Abbreviations

CDC: Centers for Disease Control and Prevention
D&I: Dissemination and Implementation
ERIC: Expert Recommendations for Implementing Change
PDSA: Plan, Do, Study, Act
PRIDI: Pragmatic, Rapid, and Iterative Dissemination & Implementation
PRISM: Practical, Robust Implementation and Sustainability Model
RE-AIM: Reach, Effectiveness, Adoption, Implementation, and Maintenance
